# Three-dimensional simulations of embolic stroke and an equation for sizing emboli from imaging

**DOI:** 10.1038/s41598-023-29974-2

**Published:** 2023-02-21

**Authors:** James P. Hague, Jonathan Keelan, Lucy Beishon, David Swienton, Thompson G. Robinson, Emma M. L. Chung

**Affiliations:** 1 School of Physical Sciences, The Open University, Walton Hall, Milton Keynes, MK7 6AA UK; 2 Department of Cardiovascular Sciences, University of Leicester, Leicester, LE1 7RH UK; 3 Department of Radiology, University Hospitals of Leicester NHS Trust, Leicester, LE1 5WW UK; 4 NIHR Leicester Biomedical Research Centre, British Heart Foundation Cardiovascular Research Centre, Leicester, LE3 9QP UK; 5 Department of Medical Physics, Leicester Royal Infirmary, University Hospitals of Leicester NHS Trust, Leicester, LE1 5WW UK; 6 School of Life Course and Population Sciences, King’s College London, Guy’s Campus, London, SE1 1UL UK

**Keywords:** Computational biophysics, Embolism, Cerebrovascular disorders, Biological physics, Medical imaging

## Abstract

Stroke simulations are needed to run in-silico trials, develop hypotheses for clinical studies and to interpret ultrasound monitoring and radiological imaging. We describe proof-of-concept three-dimensional stroke simulations, carrying out in silico trials to relate lesion volume to embolus diameter and calculate probabilistic lesion overlap maps, building on our previous Monte Carlo method. Simulated emboli were released into an in silico vasculature to simulate 1000 s of strokes. Infarct volume distributions and probabilistic lesion overlap maps were determined. Computer-generated lesions were assessed by clinicians and compared with radiological images. The key result of this study is development of a three-dimensional simulation for embolic stroke and its application to an in silico clinical trial. Probabilistic lesion overlap maps showed that the lesions from small emboli are homogeneously distributed throughout the cerebral vasculature. Mid-sized emboli were preferentially found in posterior cerebral artery (PCA) and posterior region of the middle cerebral artery (MCA) territories. For large emboli, MCA, PCA and anterior cerebral artery (ACA) lesions were comparable to clinical observations, with MCA, PCA then ACA territories identified as the most to least probable regions for lesions to occur. A power law relationship between lesion volume and embolus diameter was found. In conclusion, this article showed proof-of-concept for large in silico trials of embolic stroke including 3D information, identifying that embolus diameter could be determined from infarct volume and that embolus size is critically important to the resting place of emboli. We anticipate this work will form the basis of clinical applications including intraoperative monitoring, determining stroke origins, and in silico trials for complex situations such as multiple embolisation.

## Introduction

The consequences of emboli entering the cerebral vasculature can be devastating, and given the wide range of origins for embolic stroke, a numerical model that links disparate patterns of embolisation to stroke outcomes could be transformative for stroke research. Cardioembolic stroke (typically caused by a small number of large solid emboli) results in severe stroke presentations, and the incidence is projected to triple by 2050^1^. Large emboli can also form from other sources (e.g. extracranial atherosclerosis) and also cause severe ischemia^2^. There are several situations where large numbers of emboli can be formed, such as during cardiac surgery^3–5^; carotid surgery^6^; diving decompression^7^; and from mechanical heart valves^8^ which can lead to varying risks of neurocognitive decline and stroke. Overall, embolism accounts for approximately 40% of strokes, perhaps the largest single cause^9^.

There is a need for numerical models of embolic stroke to run in-silico trials, explore clinical scenarios to develop hypotheses for clinical studies, and provide interpretation for intraoperative monitoring. Numerical models of stroke have various potential benefits, including the potential to run in-silico “clinical” trials, explore medical scenarios (e.g. embolus fragmentation, decompression sickness and embolisation during surgical procedures), provide complementary information about the role of embolisation in parts of the cerebral vasculature that are inaccessible to imaging, and eventually to complement or replace aspects of animal models. This is important because results from animal studies of stroke may be difficult to apply to human trials^10^, recruitment to clinical trials may be challenging, and imaging techniques have limited resolution. By testing medical scenarios, computational (in-silico) models can be used to provide and assess initial hypotheses for clinical studies, potentially saving time and resources allocated to clinical trials that have no prospect of success. In this paper we demonstrate how 3D stroke simulations can be used to determine lesion volumes and locations from embolus properties, and perform an in-silico study leading to a conjectured relationship between lesion volume and embolus diameter.

Various numerical methods have previously been used to understand the motion of emboli through the cerebral vasculature. On the large vessel scale, the motion of emboli through various circle of Willis (CoW) variations has been studied using computational fluid dynamics (CFD), neglecting small vessels or embolus-flow interactions^11,12^. On the cellular scale, models including ion channels, cell metabolism and apoptosis have been used to study penumbra and lesion evolution following stroke^13–15^. Porous circulation models representing the smallest vessels have been combined with one-dimensional simulation of flow in the largest arteries of the brain (obtained from imaging, with a minimum vessel diameter of approximately 0.3 mm), within which a stroke is simulated by pinching off vessels in silico^16,17^ (in such models arterioles are treated very differently to arteries). We previously introduced a Monte Carlo method with embolus-flow interactions, but without spatial information, for predicting the severity of strokes from specific embolisation patterns^3,18–20^. Limited spatial information has been included in a similar stroke model using a tree generated with constrained constructive optimisation (details of the vasculature and its shape were not provided)^21^.

The goal of this paper is to extend our Monte Carlo simulations of stroke to include an automatically generated brain vasculature which contains vessels of all scales (from the mm scales of the largest arteries to the micron scales of the smallest arterioles) and which can mimic large cohort studies. This 3D in silico stroke model tracks emboli as they move through the vasculature to determine the locations at which they generate lesions. The Monte Carlo nature of the simulation makes it possible to run in-silico studies that mimic large cohort or population studies. As examples we run in-silico studies leading us to conjecture a simple formula for determining the diameter of the embolus causing a lesion of specific volume, and to construct probabilistic lesion overlap maps. This could form a useful basis for future ‘in silico’ clinical trials aimed at investigating the impact of clinical interventions on the incidence, volume and location of new lesions or quantifying epidemiological population-based trends in stroke imaging and outcomes over time.

## Methods

### Monte Carlo simulations

**Figure 1:**
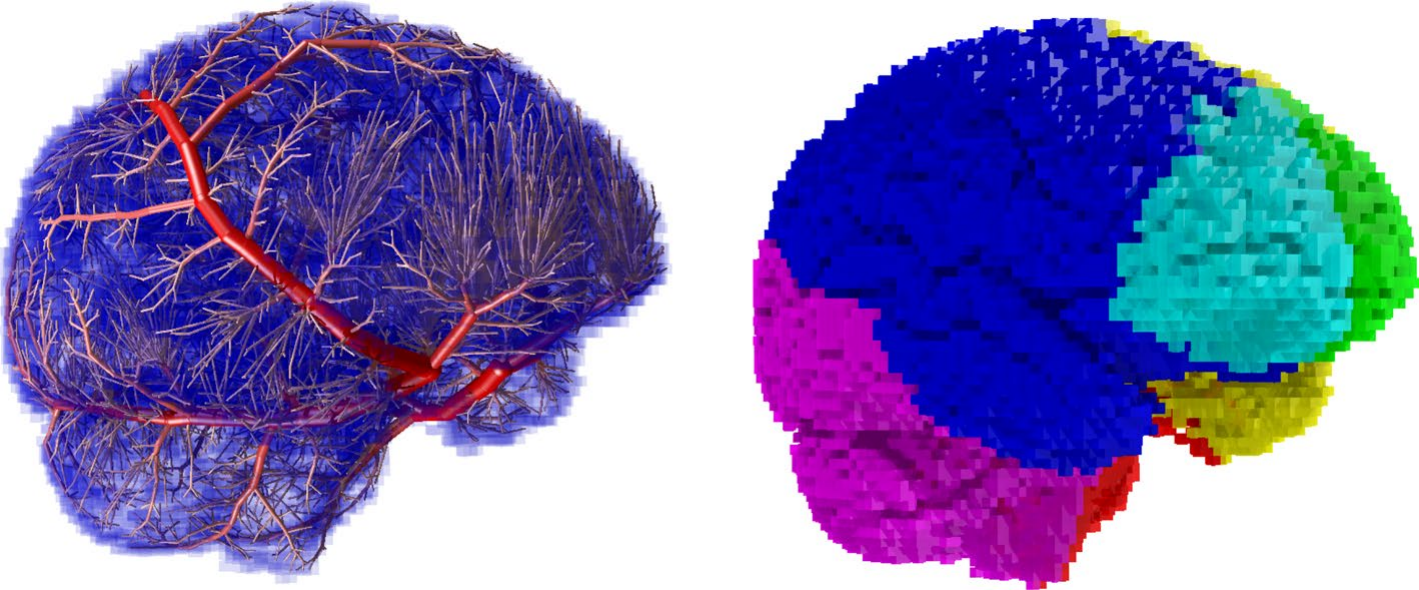
Vessels and perfusion territories in the in silico model. Green and turquoise—ACA territories. Blue and yellow—MCA territories. Purple and red—PCA territory and cerebellum.

A Monte Carlo method was used to simulate strokes. Our method was previously used with a symmetric vasculature that lacked spatial information^3,18–20^. We have extended our Monte Carlo method with an in silico vasculature^22,23^. The in silico vasculature was previously grown using the simulated annealing vascular optimisation (SALVO) technique^22,24,25^ and consists of 8192 segments representing vessels connected by bifurcations, grown according to the distribution of gray and white matter from images of a healthy individual, and with input vessel situated at the approximate location of the circle of Willis (of diameter $d_{0}$ related to diameters of middle cerebral artery, MCA, posterior cerebral artery, PCA, anterior cerebral artery, ACA and cerebellar artery, CA, via $d_{0}^{\gamma } = d_{MCA}^{\gamma } + d_{PCA}^{\gamma } + d_{ACA}^{\gamma } + d_{CA}^{\gamma }$, where $\gamma = 3.2$)^22^. In this paper, diameters of all vessels in the in-silico vasculature have been scaled such that the input vessel of the in-silico generated arterial tree is consistent with typical diameters of the major cerebral arteries (taken as $d_{MCA} = 3.1\;{\text{mm}}$ for MCA^26^, $d_{PCA} = 2.7\;{\text{mm}}$ for PCA^27^, $d_{ACA} = 2.6\;{\text{mm}}$ for ACA^28^ and $d_{CA} = 1.5\;{\text{mm}}$ for CA^29^), leading to $d_{0} = 4\;{\text{mm}}$. We have symmetrized the vasculature about the sagittal plane to create a 16,384 segment tree. The generated vasculature contains three major arteries, approximating the MCA, PCA and ACA perfusion territories. Figure 1 shows the computationally generated vasculature alongside its resulting perfusion territories^22^. We note some differences between the perfusion territories of the in silico vasculature and the human brain. In the in silico model, PCA and cerebellar territories are fused and the MCA territory is larger than typical. In terms of in-silico trials, this is not expected to cause problems, however a closer reproduction would be needed to perform inverse problems. We note a large branch from the in-silico PCA that supplies a region of the cerebellum and may act as a proxy for the inferior cerebellar arteries. To each leaf (terminal) vessel of this symmetrized vasculature we add a symmetric vascular tree with 32 elements and bifurcation exponent $\gamma = 3.2$, such that the minimum vessel diameters are 14.9 microns. The root vessels of the two hemispheres are joined at a single input for convenience. Such a vasculature has vessel sizes on all scales down to the capillary scale, and the ability to treat the metabolic requirements of gray and white matter separately. Such a vascular tree is consistent with a complete circle of Willis, which applies to approximately 50% of the population (CoW completeness can be determined by scan).

Into this tree we place simulated emboli which move until they either exit the vasculature or block a vessel according to the rules in Ref.^18^. Since it is not possible to make full fluid dynamical simulations to follow individual emboli through thousands of vessels, the complexity of the fluid dynamics is replaced by a probabilistic approach: At each bifurcation, an embolus passes down one of the two branches with a probability decided by the relative flow in each direction according to a Monte Carlo scheme, with the probability of the embolus flowing into branch A, $p_{A} = \tanh \left( {\sigma \tanh^{ - 1} \left( {2 f_{A} /(f_{A} + f_{b} } \right) - 1} \right) + 1)/2$, where $f_{A}$ is the flow in branch A, $f_{B}$ the flow in branch B^19^, unless (a) one of the branches has a smaller diameter than the embolus, in which case it travels down the larger branch (which we consider more likely due to forces on the embolus from the flow) or (b) both branches have a smaller diameter than the embolus, in which case the parent vessel is obstructed. The parameter σ varies according to the relative size between vessels and emboli. For emboli that are much smaller than the vessels in the bifurcation, *σ* = 1 is appropriate as small particles are carried proportionally to the flow. For emboli that approach the vessel size, σ can be larger, and this leads to a sigmoid function and for large σ, a step function, where emboli are compelled to move into the vessel with the largest flow. In this work, $\sigma = 1$, unless otherwise stated. Treating the probability that emboli travel into a specific bifurcation according to the relative flow is a standard approximation that has been widely used and validated experimentally^19,20,30,31^. Modelling of embolus paths through bifurcations as probabilistic reflects the complexity of fluid dynamics, which is highly sensitive to initial conditions. In simulations, for example, emboli that start in similar positions within an arterial lumen can travel down either vessel for all but the simplest bifurcation geometries, a situation that is further complicated by helical and pulsatile flows^32^.

Leaf vessels of the whole tree are monitored for lack of flow (ischaemia), and a lesion is deemed to have formed if no flow has been received for a simulated period of τ = 4 h. We explore two embolus dissolution rates: a diameter reduction rate, Δ, of 0.533 mm per 24 h (5 days for a 2.67 mm diameter embolus to dissipate) and a reduction of 0.267 mm per 24 h (10 days for a 2.67 mm diameter embolus to dissipate). Results from the model are mostly insensitive to such large differences in dissolution rate for all but the smallest emboli, the key factor being that the dissolution time for a large embolus is much greater than the timescale for ischaemia. By running the model with various embolus sizes and Monte Carlo sequences, we compute in-silico studies mimicking large cohort studies.

### Simulation image classification

LB reviewed 50 simulation images, provided in random order of size, and classified them according to whether they would be likely to be observed in a clinical setting (follow up clinic) and assigned a characterisation of ‘yes’, ‘no’ or ‘rare’. LB was blinded to embolus size. Results were then binned into histograms according to embolus size.

### Image selection

Meetings were undertaken by specialists in neuroradiology (DS), stroke and geriatric medicine (TGR, LB). The Anatomical Tracings of Lesions After Stroke (ATLAS) dataset, R1.1 (a large open access database of 304 manually segmented T1-weighted MRI stroke images^33^) was reviewed by one researcher (LB). Typical examples of anterior, middle and posterior territory embolic stroke were selected from the database. Manual segmentation is currently the gold standard method for segmenting brain lesions on T1-weighted imaging. Images were selected for this study by consensus discussion between the three Clinicians to ensure they were representative of embolic lesions and to confirm the perfusion territory affected. Cases were chosen from the ATLAS data-set that were representative of the following radiological characteristics associated with embolic stroke: multiple vascular territories and or hemispheres affected, cortical, cerebellar, or brainstem location, > 1.5 cm diameter lesions^34,35^. These images were reviewed at a consensus meeting held between LB, TGR and DS and the best examples for each territory were selected for replication by the stroke simulation based on the same criteria. Initial simulated images were compared at a further consensus meeting against the ATLAS dataset. Qualitative feedback was obtained with regard to the size of the simulated perfusion territories and limitations of the model and the number of sample simulations was increased to 1000 before matching to the clinical images, resulting in a final set of simulations for review by the consensus group. Clinical feedback from the original simulated images was used to identify matching images from a larger number of simulations.

### Power law fit

A power law curve (with offset) approximation to the percentage of the vascular tree occluded (% infarct volume) in the simulated strokes, $\% {\text{infarct}}\;{\text{volume}} = a\left( {\left( {d - d^{\prime}} \right)/d_{0} } \right)^{b}$, was fit to the data using the ‘fit’ function of Gnuplot 5.4^36^, with a, b and d’ coefficients as the fit parameters. In this function, *a* is a percentage (which should be close to 50% so that when *d* = *d*_*0*_, a whole hemisphere is blocked), *b* is an effective bifurcation exponent (which should be close to 3.2), and *d’* represents the change in embolus diameter during the time it takes a lesion to form, $d^{\prime} \approx \tau \Delta$. The fit function of Gnuplot uses a nonlinear least squares algorithm (the Marquardt-Levenburg algorithm) allowing this function to be fit directly to the data.

## Results

**Figure 2:**
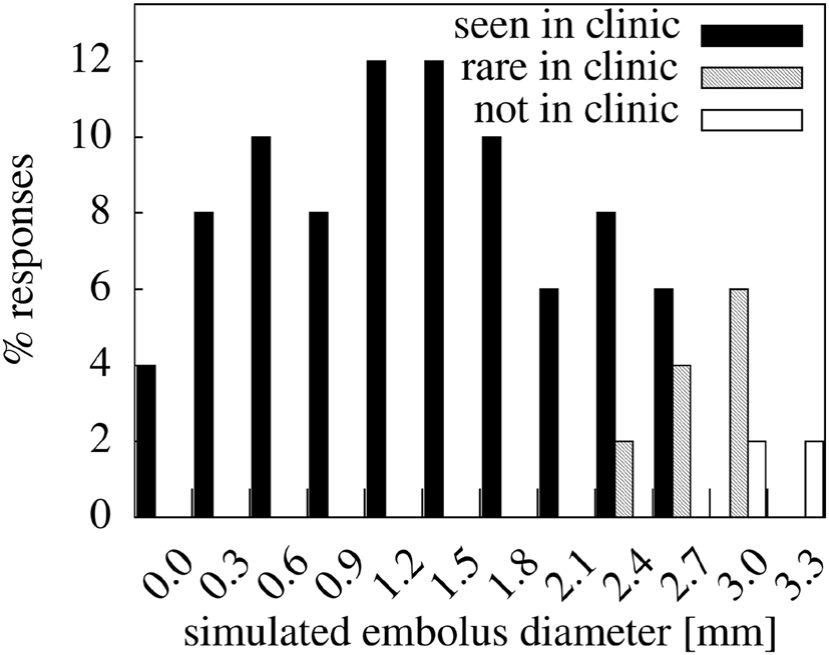
Clinical classification of simulated strokes. A clinician (LB) was given images of the in-silico lesions and asked to assess them as to whether they resemble cases relating to follow-up clinic “likely to be seen in clinic”, “rare in clinic” or “not seen in clinic”. This may suggest the maximum embolus diameter commonly seen in follow-up clinic is ~ 2.85 mm.

The majority of stroke outcomes from the model for realistic embolus sizes appear clinical. Figure 2 shows assessment of simulated strokes by a Clinician as to whether they resembled lesions seen in a follow up clinic, finding that lesions in the in-silico model resembling those in clinic or rarely seen in clinic are typically caused by emboli with diameters of 2.85 mm or less. 100% of simulated lesions caused by emboli with diameters < 2.25 mm were rated as “typically seen in clinic”. Simulated lesions identified as similar to those typically seen in clinics related to a maximum embolus diameter of 2.85 mm. Some in silico lesions caused by large emboli of between 2.25 and 3.15 mm diameter were classified as rarely seen in clinic, and accounted for ~ 4% of simulated lesions. Most in silico lesions generated by emboli of 2.85 mm or greater were not consistent with those typically seen in clinic (as expected).

**Figure 3:**
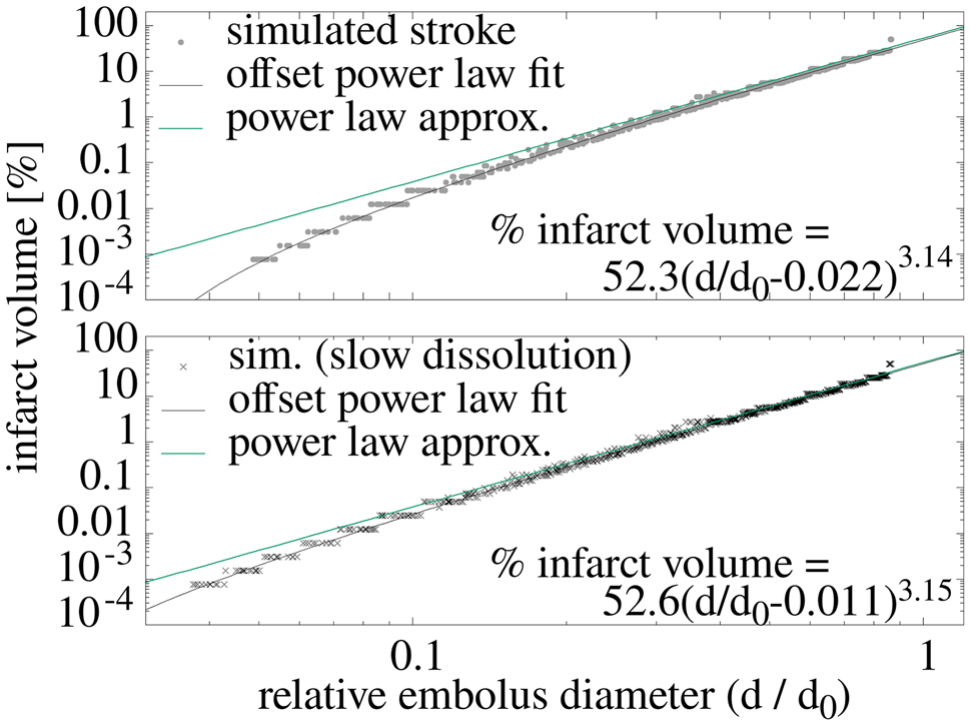
Volume of in silico infarcts as a proportion of brain size increases with embolus diameter according to a power law. Both the infarct volume and embolus diameter are plotted on logarithmic scales to demonstrate this power law relationship. Lesion volumes were calculated for two different dissolution rates, and results for large emboli were insensitive to this parameter.

Simulations featuring emboli of varying size entering the in silico vasculature show that infarct volume grows rapidly with embolus diameter, following a power-law distribution as shown in Fig. 3. The infarct volume as a percentage of total brain volume fits well to a power law (black line labelled, ‘offset power law fit’) given by:

1 $$\% {\text{ infarct}}\;{\text{volume}} = a\left( {\left( {d - d^{\prime}} \right)/d_{0} } \right)^{b}$$

where the diameter of the embolus is *d.* For both dissolution rates, the dimensionless parameters, a and b obtained from fits are consistent with each other, having the average $a = 52.4 \pm 0.4$, and $b = 3.145 \pm 0.01$. This result is provided as a percentage of total brain volume and the ratio $d/d_{0}$ so that the expression is independent of variations in individual brain volume and vessel diameters. The parameter d’ depends on the dissolution rate. For the faster dissolution rate, the fit value d’/d_0_ = 0.0224 + /− 0.0005, and for the slower rate, 0.0105 + /− 0.0005, both of which are consistent with $d^{\prime} = \tau \Delta$. Equation (1) with d’ set to 0 is also shown (turquoise line, ‘labelled power law approx.’) and approximates the limit of very slow dissolution rates.

Equation (1) can be inverted to determine the diameter of an embolus causing an infarct as:

2 $$d = d_{0 } \times [\% {\text{infarct}}\;{\text{volume}} / a]^{1/b} + \tau \Delta$$

(we note that this equation provides a conjecture/hypothesis and that clinical studies would be needed to confirm this relationship). Variations in the infarct volume occur because emboli of similar sizes may travel different paths due to the Monte Carlo nature of the simulations, and were typically less than a factor of two. Lesion volumes were calculated for two different dissolution rates, and results were insensitive to this parameter. We note that for ischemic non-embolic stroke (e.g. atherosclerotic stroke), the blockage develops in-situ. In such cases, we expect that Eq. (2) (setting d’ = 0) would hold for the relation between lesion size and diameter of blocked vessel, although the frequency with which lesions of certain sizes appear may vary between embolic and non-embolic ischemic strokes.

**Figure 4:**
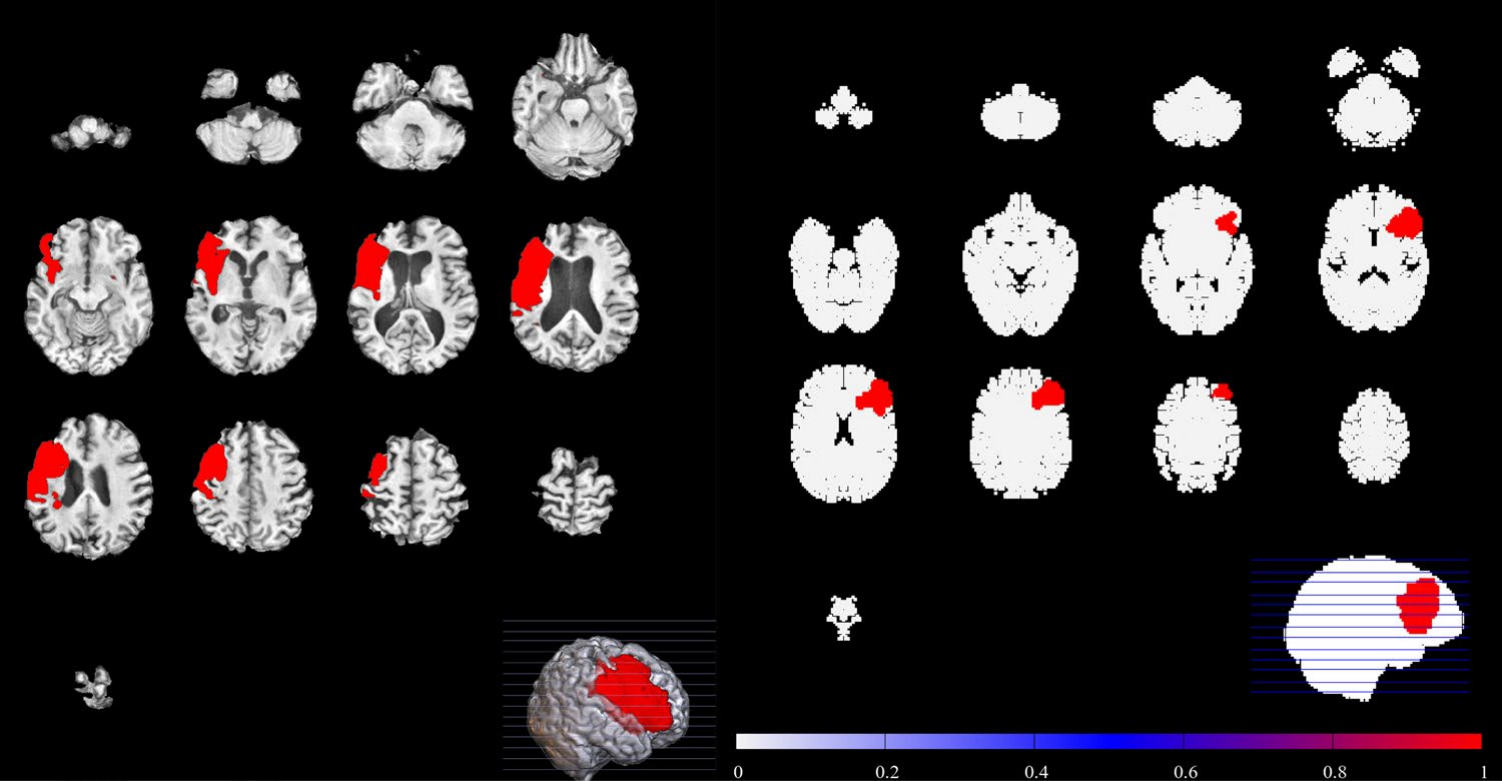
MCA stroke example extracted from the ATLAS database (left), compared to an example stroke simulation (right). Smaller MCA strokes were well replicated in the simulation images.

**Figure 5:**
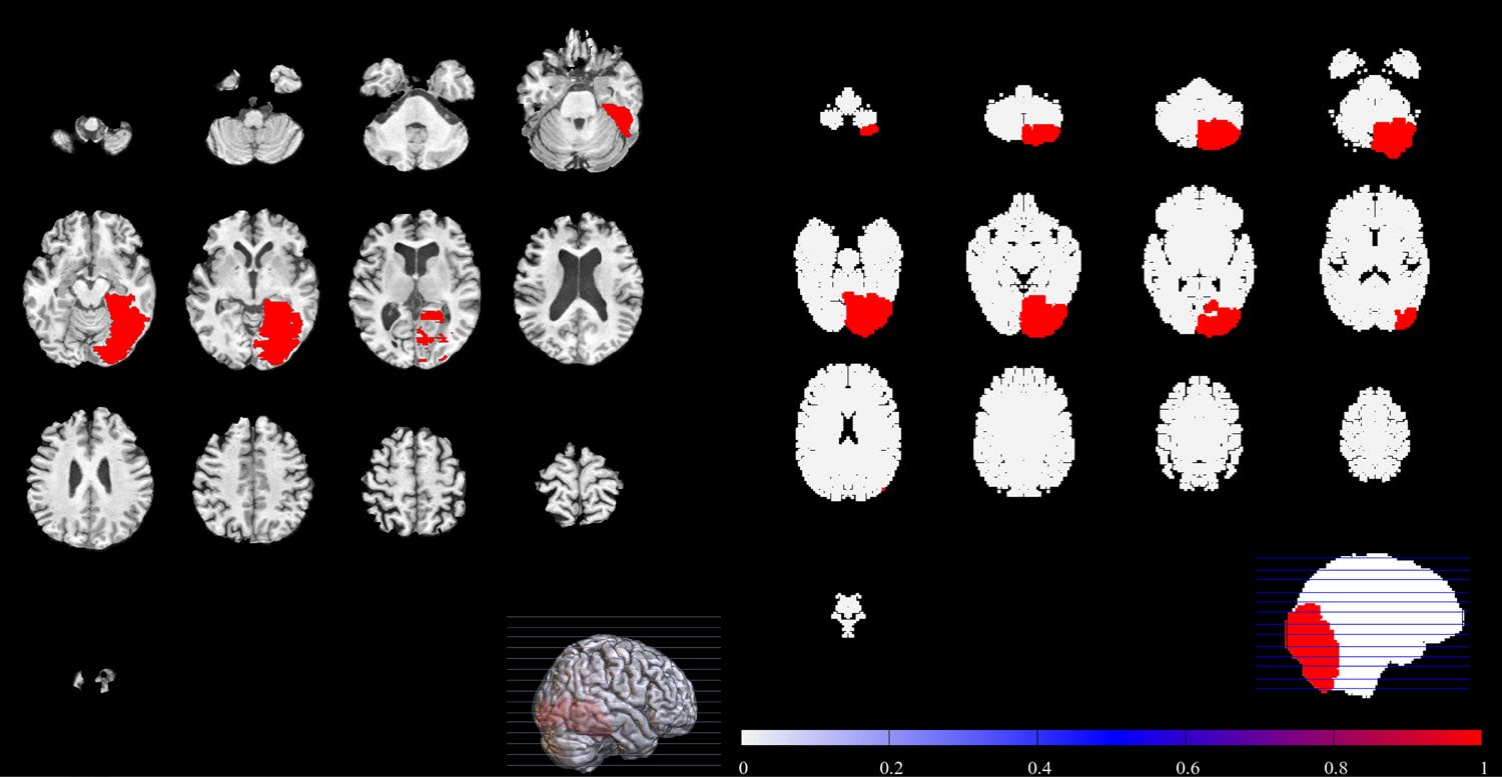
PCA stroke example extracted from the ATLAS database (left), compared to an example stroke simulation (right). PCA territory lesions were well reproduced by the simulation.

**Figure 6:**
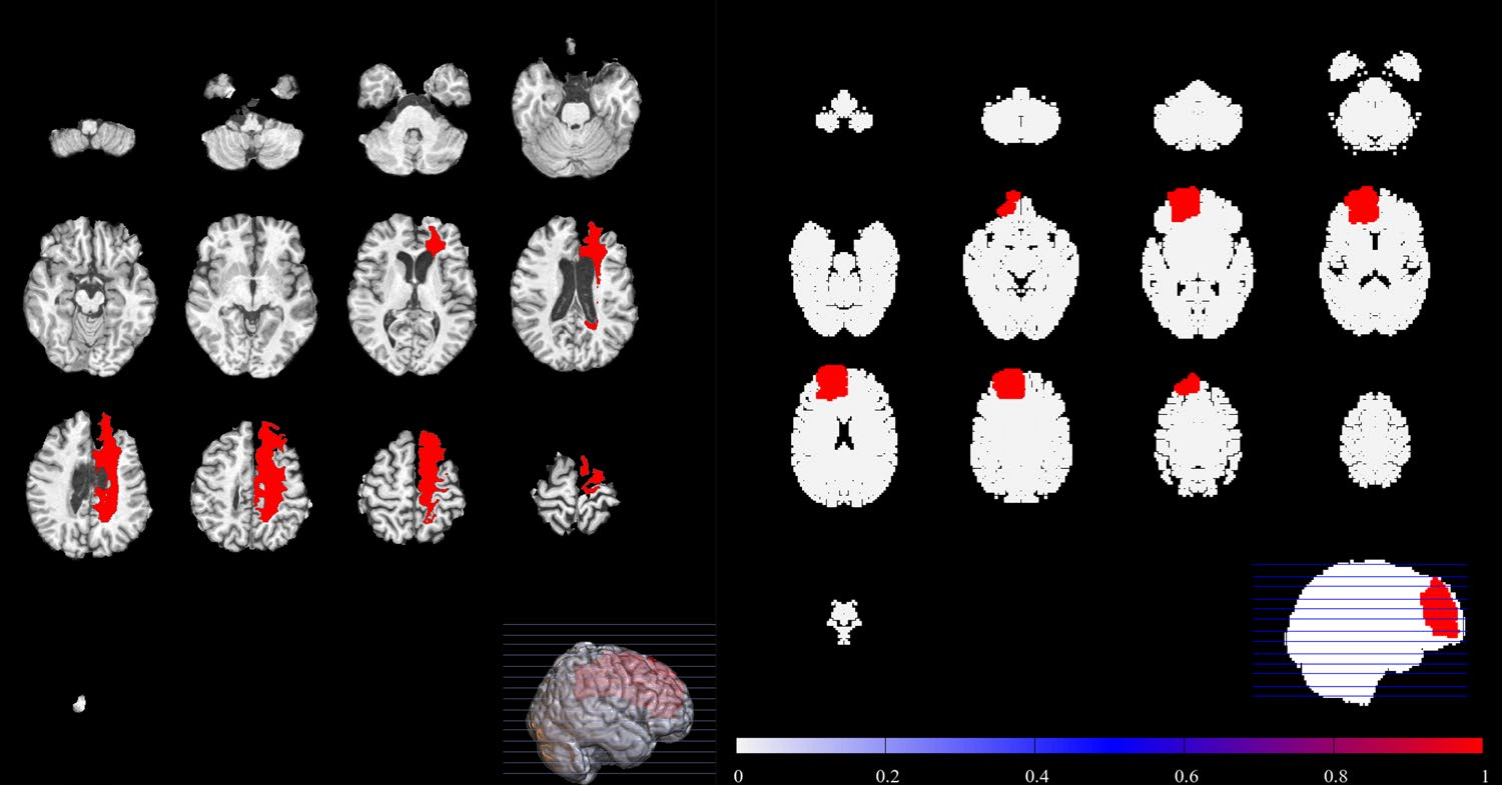
ACA stroke example extracted from the ATLAS database (left), compared to an example stroke simulation (right). The location is broadly similar. However, the ACA perfusion territory extends superiorly from anterior to posterior in the ATLAS example than in our simulations.

Specific instances of stroke are presented in Figs. 4, 5 and 6. The left hand panels of Figs. 4, 5 and 6 show examples of stroke from the ATLAS database, and the right hand panels show examples of similar strokes generated by our computational model. In each computational case, a single embolus was introduced into the vascular tree and allowed to propagate through the arterial tree. The results have been compared with scans from the ATLAS database that were confirmed to be representative of embolic stroke by the specialist clinicians (TGR, DS, LB). Strokes occurred in the MCA (Fig. 4) PCA (Fig. 5) and ACA (Fig. 6) territories of the in silico vasculature. Qualitative feedback from the clinical consensus meeting was that: (1) Smaller MCA strokes (shown in Fig. 4) were well replicated in the simulation images. The largest MCA strokes involved other perfusion territories (ACA and PCA), probably due to the slightly different shape of the territories in the computational vasculature (Fig. 1). (2) PCA stroke (shown in Fig. 5) was well replicated in the simulated images. (3) We note that ACA strokes (example shown in Fig. 6) were rare in the simulations (as is the case clinically). ACA stroke in some of the simulated images included an area affecting the MCA region which was not typically seen clinically and may be due to the difference between perfusion territories of the in silico and human vasculatures. (4) Borderzone infarcts were less well replicated in terms of location and shape of the infarct. These differences in territory likely match those of the in-silico vasculature, and we do not expect them to lead to major problems if used for hypothesis generation (discussion on potential improvements can be found in the next section). Taken in the context of Fig. 2, there is good reproduction on increasing embolus size, until emboli become so large that they block multiple territories.

**Figure 7:**
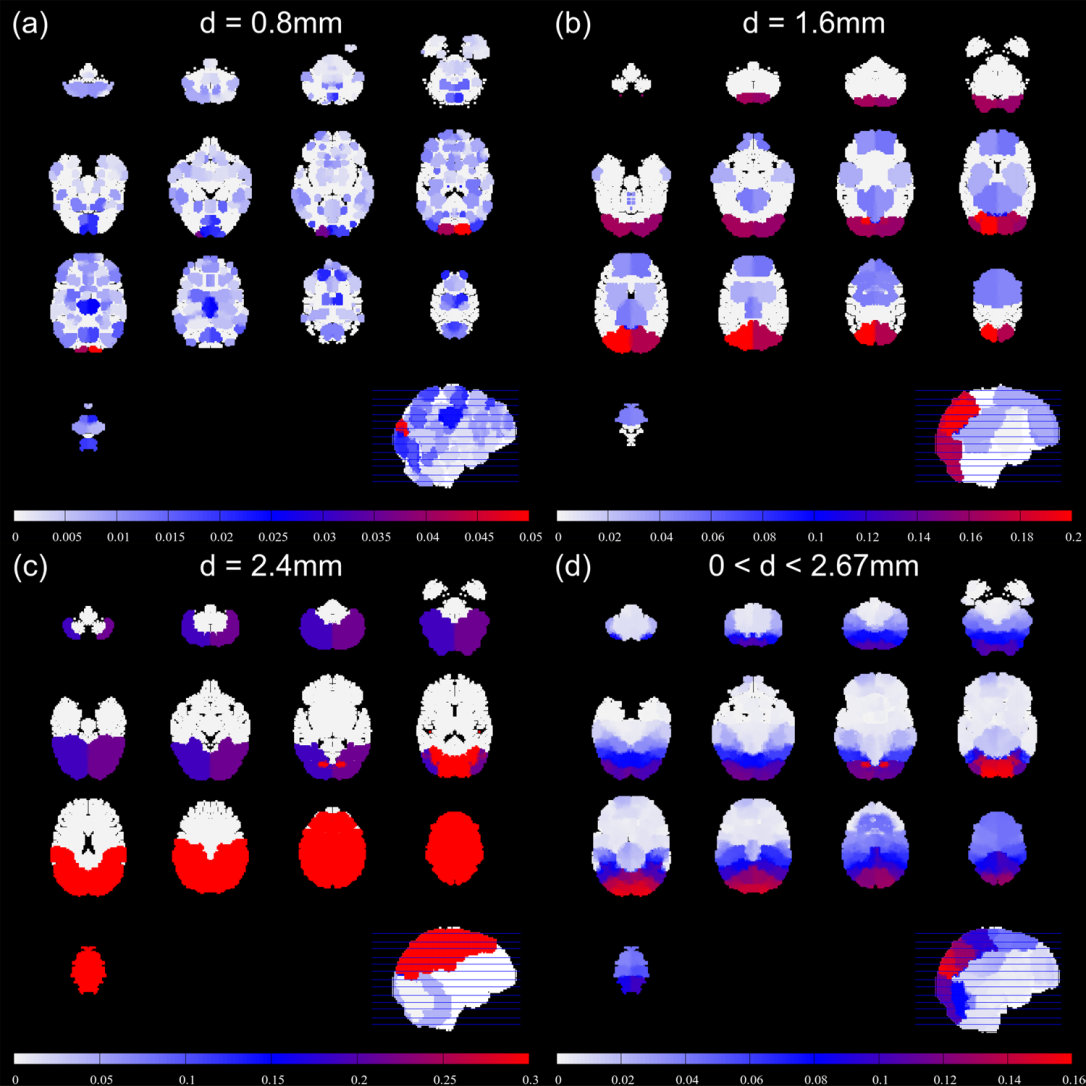
Maps showing the probability that emboli of particular sizes will arrive at a location in the brain. Small emboli are evenly distributed, whereas large emboli preferentially come to rest in the MCA territory of the in silico model. When receiving a distribution containing emboli of various sizes, emboli arrive preferentially in the posterior + superior MCA territory of the in silico model, but lesions are also common in the posterior and superior parts of the brain. Here $\sigma = 1.25$.

Probabilistic lesion overlap maps showing spatial distributions of lesions resulting from different size distributions of emboli are shown in Fig. 7. These maps provide an estimate of the risk that an embolus causes a lesion at a specific brain location. The spatial distribution of lesions was found to be highly dependent on embolus size. Figure 7 shows the probability of lesions for (a) emboli of 0.8 mm diameter, (b) emboli of 1.6 mm diameter, and (c) emboli of 2.4 mm diameter. Of these three sizes, the 0.8 mm diameter emboli lead to the most spatially homogeneous distribution of lesions across the brain affecting multiple perfusion territories. 2.4 mm diameter emboli preferentially block the MCA territory. 1.6 mm diameter emboli preferentially reach the MCA and PCA territory, with some ACA territory strokes. Thus, the size of emboli critically influences their trajectories through the vasculature and indicates a preference for larger emboli traveling into the MCA territory, leading to larger lesions occurring in the MCA territory.

When a flat distribution featuring equal numbers of emboli of differing sizes, up to a 2.67 mm maximum diameter is introduced to the in silico vasculature, obstruction of the posterior MCA territory was most common, with further blockages occurring in both the PCA territory, and the remainder of the MCA territory, but with fewer lesions in the ACA territory, as shown in Fig. 7d. A flat distribution is used because the true distribution of embolus sizes relating to strokes seen in clinics is not known. In the ATLAS data-set, MCA infarcts are most probable. This is also true in our in silico vasculature, although the most probable location in the simulations was found to be towards the superior-posterior region of the MCA territory. Our simulations have large numbers of lesions in the PCA territory. ACA infarcts were rare in both the ATLAS dataset and our simulations.

## Discussion

In this study, we demonstrated that 3D Monte Carlo simulations of embolic stroke can be used to make predictions about the size distributions and locations of lesions due to specific patterns of embolisation. There are several advantages to stroke modeling in this way. Firstly, spatial information is available enabling direct comparison with radiological images. Secondly, in silico clinical trials could be conducted. Thirdly, the simulations require no prior knowledge of the patient’s anatomy or status other than that already contained within standard brain imaging data. Clinical feedback indicates that simulated lesions were realistic for embolus sizes below 2.85 mm, comparable with the MCA diameter.

By way of example, we have determined an expression to relate embolus size to lesion volume. Thus, we have shown how large in-silico trials can be used to develop a hypothesis. Clinical studies need to be carried out to identify if the information obtained from this expression is useful and accurate for clinical outcomes. We have also shown how probabilistic lesion overlap maps can be generated using the numerical model. Such maps could be useful as input to Bayesian techniques. We are not aware of any routine approach to sizing the embolus that caused a stroke, so our approach has been successful at identifying and filling a gap. This is the first step to solving the inverse problem, by taking the lesion and grey and white matter distribution, as well as 3D MR angiography, to predict the likely origins of emboli based on the patient’s observed pattern of lesions.

As the sophistication of in silico models improves, there is an opportunity to complement or replace animal models and to extend our model based on cellular biology to perform detailed in silico clinical trials. Experimental models of stroke are often animal based, but results may be difficult to apply to humans^10^. So complementary numerical models could improve decisions on which hypotheses to take forward into clinical studies.

Another application of the stroke simulations presented here relates to predictive simulation during intraoperative monitoring and multiple embolisation events. Monte Carlo methods are ideal for simulating showers of solid and gaseous emboli during cardiovascular interventions, such as cardiac or carotid surgery, cardiac ablation and stent deployment^3,18–20^. In these settings, embolic events can be detected using intraoperative monitoring and could be directly incorporated into a 3D stroke simulation to predict the impact of emboli on brain tissue perfusion, in a similar manner to Ref.^3^. We will discuss the modeling of multiple embolisation in a future publication.

A strength of our simulation method is inclusion of vessels on all length scales, embolus-flow interactions and tracking of embolus paths through the tree. Further strengths include comparison with a large real-world image database which used manual lesion segmentation, with all scans and simulation images reviewed by a consultant neuroradiologist before inclusion in this study (since images in ATLAS were not always segmented by neuroradiology experts).

Future extensions to the model include: Enhancements to the vasculature, using e.g. vasculature taken from MRI imaging augmented using the SALVO method, simulations differentiating between gaseous and solid emboli, better representation of the CoW and personalisation. Refinements to CoW representation would improve stroke simulations for cases where embolus sizes exceed the diameters of the largest vessels exiting the CoW, so that lesions do not cross ACA, PCA and MCA boundaries. Personalization would enhance the in-silico vasculature by seeding the vascular growth algorithm based on patient-specific time-of-flight MR angiography data, with additional arterial inputs for ophthalmic arteries, cerebellar arteries and other major vessels, and representative variants of the CoW (to account for the 50% of the population that do not have a complete CoW, including any related modifications to perfusion territories due to CoW topology). This breakthrough in modelling embolic stroke would provide a means of assessing the impact of anatomical and other variants under controlled conditions through performing simulations. Ideally databases containing acute rather than chronic stroke images would be used for comparison with simulation results.

Further downstream, we propose that Monte Carlo stroke simulations could be used to determine the cause of an acute ischaemic stroke. Personalized stroke simulations aimed at rapid diagnoses of the location and source of a stroke could be useful to distinguish cardioembolic sources from other types of stroke, facilitating rapid access to targeted treatment. This is a complex problem currently based on analysis of a range of factors including patient characteristics and risk factors, as well as neurological symptoms and imaging data (as the location, size, and pattern of infarcts often indicate likely etiology). At present there are no quantitative criteria or tools by which this is assessed, relying on the skills of an experienced Radiologist, or treating Clinician^37^. Up to 25% of strokes are currently classified as embolic of undetermined source (ESUS)^37,38^ and correct identification of atrial fibrillation (AF) is thought to occur in only 50% of eligible patients, representing a significant barrier to timely anticoagulation^37^. Rapid identification of the source of emboli, and ability to distinguish between cardioembolic and other causes of stroke, has the potential to guide clinical treatment and improve patient outcomes. Further tailoring of simulations to include patient-specific imaging data and match in silico predictions to real-world imaging, coupled with implementation of artificial intelligence (AI)-based feature extraction (segmentation) from images may be useful for automatically predicting the size, location (and potentially the source) of embolisation to improve image interpretation methods, and save neuroradiologists’ time. As a first step in this direction, we propose that Eq. (2) can be used as a tool to provide estimates of embolus size from imaging data, especially in cases where it is difficult to determine the lumen diameter for an occluded vessel (we note that further clinical study would be needed to validate the equation, and assess if such additional information improves patient outcomes). A limitation of our simulations for this application is that in silico perfusion territories reproduced by the model differ slightly from those seen in the human brain. As the simulations and their associated probabilistic lesion overlap maps (and other measures such as the correlation between the hemispheres where multiple lesions occur, or whether the lesions are subcortical) are improved, we suggest that the probability that the embolus has a particular size and type (and ultimately origin information) could be extracted by e.g. comparing imaged lesions to the probabilistic lesion overlap maps associated with different embolic sources. Additional steps in this direction should include comparison of simulation results with patient diagnosis and outcome to inform predictive diagnostics. Ultimately, machine learning methods could be used to train the model and improve its predictive capacity, and would allow the patient’s symptoms, physiology, cellular biology, and genetic or demographic data, to be incorporated with advanced image interpretation.

## Data Availability

The datasets generated and analysed during the current study are available in the Open Research Data Online (ORDO) repository, https://doi.org/10.21954/ou.rd.19682874. Software will be made available from the corresponding author on reasonable request. Requests will be considered from academic users only, and for validation purposes only due to commercial sensitivity.
